# Transcriptome Analysis Reveals the Complex Regulatory Pathway of Background Color in Juvenile *Plectropomus leopardus* Skin Color Variation

**DOI:** 10.3390/ijms231911186

**Published:** 2022-09-23

**Authors:** Feibiao Song, Lei Wang, Zihang Yang, Liping Shi, Da Zheng, Kaixi Zhang, Junlong Sun, Jian Luo

**Affiliations:** State Key Laboratory of Marine Resource Utilization in South China Sea, Hainan Aquaculture Breeding Engineering Research Center, Hainan Academician Team Innovation Center, Hainan University, Haikou 570228, China

**Keywords:** *Plectropomus leopardus*, background color, skin color, RNA-seq, melanogenesis

## Abstract

Fish skin color is often strongly affected by background color. We hypothesized that the regulatory mechanism of variations in skin color in *P. leopardus* is linked to the background color. In this study, we conducted transcriptome analysis of *Plectropomus leopardus* cultured under different background colors to compare gene expression levels and the important signaling pathways. The RNA-seq analysis yielded 26,675 known mRNAs, 3278 novel mRNAs, and 3179 differentially expressed genes (DEGs). The DEGs related to melanin synthesis were screened out. Some key melanin-related genes were identified, specifically *tyr*, *slc7a11*, *mc1r*, *ednrb*, *dct*, *tat*, and *wnt1*. These DEGs were mainly involved in melanogenesis, including tyrosine metabolism, the Wnt signaling pathway, and the cAMP signaling pathway. The expression levels of some key genes were upregulated when background color deepened, such as *α-msh*, *wnt*, and *gf*. The α-MSH/cAMP-dependent, Wnt/β-catenin, and PI3K/Akt signaling pathways were activated, resulting in the accumulation of intracellular *mitf*. *mitf* promoted melanin production by binding to the *tyr*/*tyrp1*/*dct* promoter region. In the present study, we explored the molecular mechanism underlying the darkened skin color pattern of *P. leopardus*, providing a theoretical basis for the molecular mechanism underlying pigmentation in *P. leopardus*.

## 1. Introduction

Fish skin is colorful and patterned and is closely related to many behaviors, such as camouflaging, environmental stress, and courtship. Fish skin color is under multiple selection pressures and is one of aquaculture’s most important commercial traits. Skin color has been classified into morphological and physiological types, which interact with genetic and environmental factors [1,2,3,4,5]. Therefore, exploring the evolutionary mechanism underlying complex and diverse pigmentation in fish is an important topic in evolutionary biology [6]. Fish are ideal models for studying skin color variations because of the phenotypic plasticity of skin color and the diversity of pigment cells [6,7].

Culture tanks offer numerous advantages, whether configured as permanent or temporary structures, and tank color significantly affects the physiology of aquatic animals [8,9]. Tank color can be readily manipulated during the production stage of aquaculture. Moreover, white, blue, or black color tanks are usually used, but the rationale behind their use is seldom explained [8]. Adaptation to the background that occurs in many teleosts is a physiological response to photic and color variations, including aggregation and dispersion of pigments triggered by neural stimuli [10,11,12]. Fish could change their skin color in response to photic conditions and the brightness or hue background [5,13].

Color change is closely associated with the endocrine and neuroendocrine systems of the hypothalamus–pituitary axis [11,14]. The α-melanocyte-stimulating hormone (*α-msh*) and melanin-concentrating hormone (*mch*) are two peptide hormones controlling body color with opposite functions in the chromatophores of fish [11,14,15]. The *mch* stimulates the aggregation of pigments in the chromatophores and induces a pale body color. In contrast, the α-MSH disperses the pigments in the chromatophores and darkens skin color [13]. The precursor protein proopiomelanocortin (*pomc*) is secreted from glandular cells in the pituitary, and proteolytic cleavage of *pomc* generates α-MSH [13]. Many studies have indicated that skin color changes in aquatic animals caused by background color occur due to changes in the expression levels of *mch*, *α-msh*, *pomc*, and other genes. Skin color and the expression level of *pomc* are affected by background color in *Carassius auratus*, suggesting that *mch* and *α-msh* are involved in regulating skin color and are affected by background color [16,17]. *Oncorhynchus mykiss* have their brightest body colors and the highest *mch* expression levels in white tanks, and *pomc*-a and *pomc*-b are more highly expressed in black tanks, as skin color is affected by tank brightness [18]. Similarly, the expression of *mch* mRNA is higher in red tilapia tanks with a white background than in black tanks (*p* < 0.05). In contrast, *pomc* mRNA levels are higher in black tanks than in white tanks (*p* < 0.05) [19].

Many signaling pathways regulate fish skin color variations, and transcriptomics research has provided data on fish coloration and pigmentation [20]. For example, the Illumina platform has been used to sequence Xingguo red carp and Yellow River carp. According to the gene pathway analysis, the Wnt and MAPK signaling pathways likely affect the skin pigmentation process [21]. Comparative transcriptome analysis has indicated that the Wnt/β-catenin pathway is important in regulating melanin synthesis and melanophore differentiation in red tilapia [6]. Research on melanin synthesis is relatively advanced, and multiple pathways are involved [20]. Previous studies have shown that four signaling pathways—α-MSH/cAMP-dependent, MAPK, PI3K/Akt, and Wnt/β-catenin—are key regulatory pathways in melanogenesis, which activate or depress melanin synthesis [6,20].

The leopard coral grouper (*Plectropomus leopardus*) is a valuable marine fish in the subfamily Epinephelidae. Moreover, this species is an important resource for intensive industrial farming in recirculating aquaculture systems due to its high commercial value and broad market prospects. The fish’s skin color becomes black, brown, and red under intensive culture; however, the molecular mechanisms underlying these diverse color patterns are poorly understood [22]. In the present study, we conducted transcriptome analysis of *P. leopardus* cultured under different background colors to examine the gene expression levels and signaling pathways. In particular, the molecular mechanism underlying the dark skin color pattern of *P. leopardus* was explored, providing a theoretical basis for the molecular mechanisms of underlying *P. leopardus* pigmentation.

## 2. Results

### 2.1. The Types of Chromatophores in P. leopardus under Different Backgrounds

**Figure 1:**
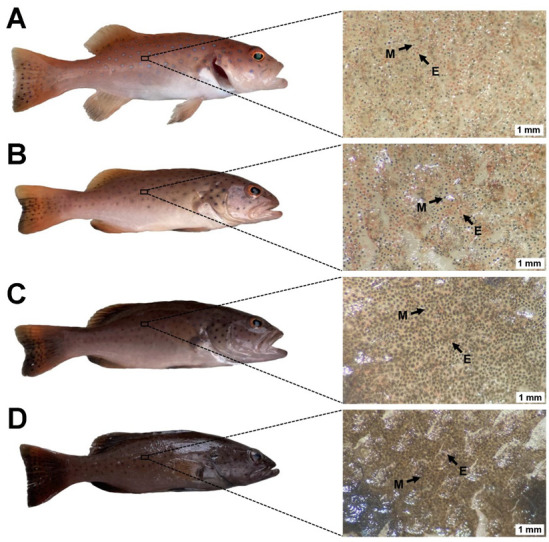
Skin color phenotypes under different backgrounds and the chromatophore types in *P. leopardus*: (**A**) initial, (**B**) white background, (**C**) blue background, (**D**) black background; the letter E marks erythrophores, and the letter M marks melanophores.

The skin colors of the *P. leopardus* reared in this study are shown in Figure 1. At the end of the experiment, the white group maintained a relatively red skin color compared to the initial group. Nevertheless, the skin color of the fish in the blue and black groups blackened. Optical microscopy showed that melanophores and erythrophores were present in the skin of all groups. The number and cell area of the melanophores increased as the background color darkened. Some erythrophores with a dispersed distribution were observed. Furthermore, the erythrophores were obscured by many crowded melanophores; thus, less red color was observed under gross inspection.

### 2.2. Overview of RNA Sequencing Data

A total of 567,815,660 raw reads were produced from 12 cDNA libraries of *P. leopardus* skin. After filtering the data, the clean read count was 79.26 Gb. The results revealed that more than 90% of clean reads were mapped onto the *P. leopardus* reference genome. The average proportion of sequences with unique positions mapped to the reference sequence was 80.25%, and approximately 40% of the reads were mapped to the positive and negative strands of the genome (Table S2).

### 2.3. Characterization of the Skin mRNAs

**Figure 2:**
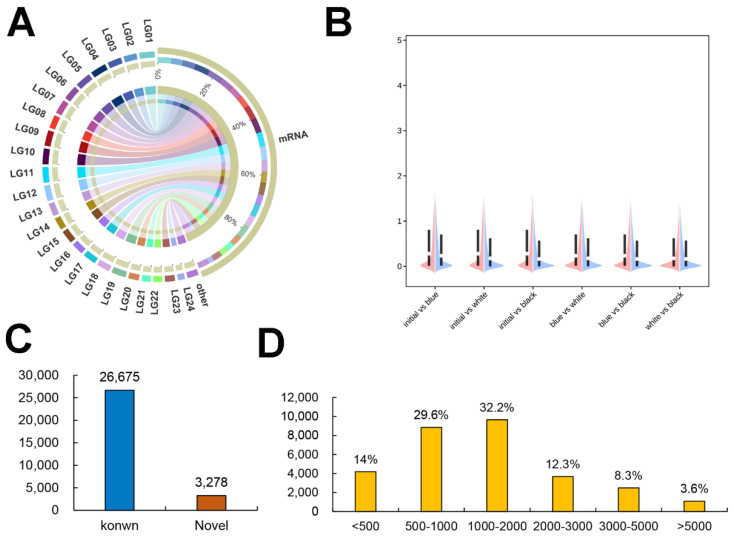
Characterization of skin mRNAs in different groups. (**A**) The distribution of mRNA on the chromosomes. (**B**) Violin plot of mRNA expression level in different groups. (**C**) Number of mRNAs. (**D**) Length of the mRNAs.

Figure 2A shows that these transcripts were randomly distributed on the *P. leopardus* chromosomes (Table S3). Violin plot analysis of the different groups on the FPKM values of the transcripts showed different distributions between the two groups (Figure 2B). After a stringent filtering process, 26,675 known and 3278 novel mRNAs were obtained (Figure 2C). Statistics on the structural characteristics of the mRNAs showed that over 60% had lengths of 500–2000 bp (Figure 2D).

### 2.4. DEG Analysis among the Four Groups

**Figure 3:**
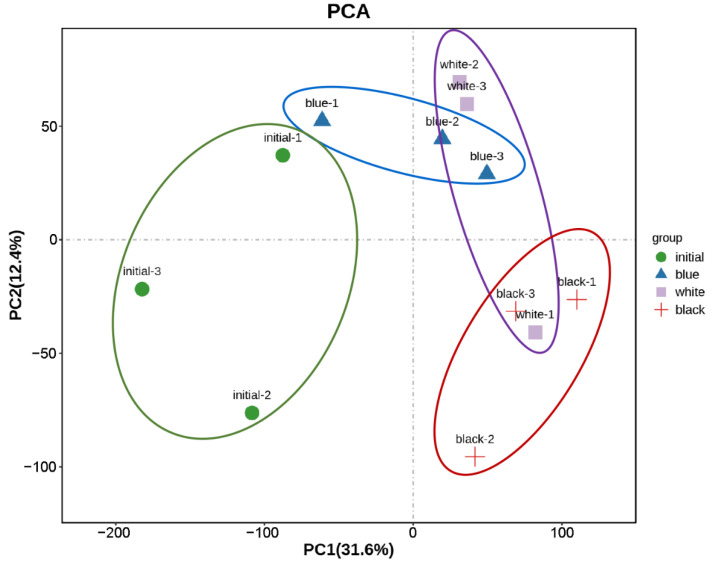
PCA analysis of the 12 datasets.

The initial and black groups were considered extreme phenotypes, and the relatively white and blue groups were intermediate phenotypes. A principal component analysis (PCA) of the 12 datasets (Figure 3) showed that the differences between the two groups were more significant than the intragroup differences, and the white and blue groups shared the closest relationship. One sample (white-1) appeared to be an outlier. We speculate that this sample originated from errors in sequencing or experimental fish status; consequently, the sample was omitted from subsequent data analyses.

**Figure 4:**
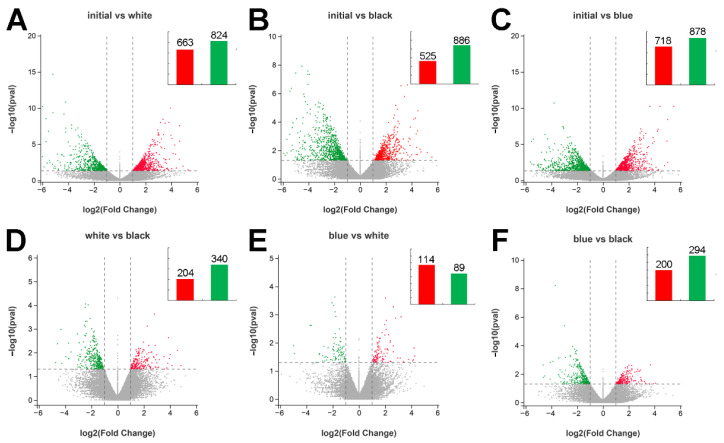
Differentially expressed mRNAs in different groups. (**A**) Volcano plots of initial vs. white. (**B**) Volcano plots of initial vs. black. (**C**) Volcano plots of initial vs. blue. (**D**) Volcano plots of white vs. black. (**E**) Volcano plots of blue vs. white. (**F**) Volcano plots of blue vs. black. Red: upregulated genes; Green: downregulated genes; Grey: no significant differentially expressed genes.

Six contrasting groups were generated by all possible combinations of the four experimental groups. The specific numbers of up- and downregulated DEGs in the six contrasting groups are shown in Figure 4. As shown in Figure 4, more DEGs were found in the initial vs. white, initial vs. black, and initial vs. blue groups (Figure 4A–C) than in the white vs. black, blue vs. white, or blue vs. black groups (Figure 4E). Notably, there were fewer upregulated genes than downregulated genes in the light-skinned group vs. the dark-skinned group. The different gene expression profiles of the six contrasting groups are shown in Table S4.

**Figure 5:**
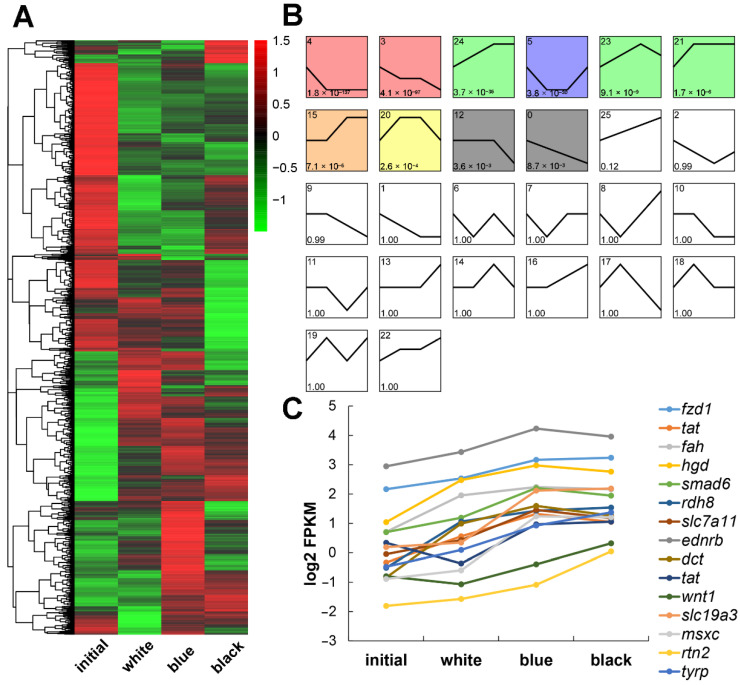
Hierarchical cluster analysis and expression profile of DEGs in *P. leopardus* of different skin colors. (**A**) The heatmap represents the log2 fold changes (*p* < 0.05) of DEGs. (**B**) Expression patterns of DEGs (The colored box indicated significant difference, *p* < 0.05). (**C**) The specific upregulated DEGs related to the skin color from red to black.

We focused on the expression patterns in the four groups and not just the pairwise comparison to identify genes that could be included in *P. leopardus* skin color darkening. Here, we arranged the skin color from red to black as initial, white, blue, and black. The heatmap in Figure 5A shows the DEGs collected from the six contrasting groups. Blue and red represent low and high expression levels, respectively. The 3179 DEGs were grouped into 26 clusters according to their expression patterns, and the line charts are shown in Figure 5B. The upregulated genes in clusters 15, 21, and 24 correspond to gradual changes in skin color, so they may be potentially related to black skin formation. The downregulated genes in clusters 0, 3, and 12 could be potentially related to red skin formation. As shown in Figure 5C, some genes in the upregulated clusters were related to melanophores and melanin, such as *slc7a11*, *ednrb*, *dct*, *tat*, and *wnt1* [20]. All 3179 DEGs are shown in Table S5.

### 2.5. GO and KEGG Enrichment Analyses of the DEGs

DEGs from the six contrasting groups were generated and annotated by the GO analysis, and Figure S1 shows the top 20 GO terms in every group. At a *p* < 0.05 cutoff, 1087 GO terms were enriched in the six DEG sets (Table S6). Most genes in all six sets were involved in the biological processes category, followed by molecular functions and cellular components (Table S6). The DEGs in biological processes were associated with biosynthetic and metabolic processes, such as peptide biosynthetic processes (GO: 0043043), amide biosynthetic processes (GO: 0043604), peptide metabolic processes (GO: 0006518), cellular amide metabolic processes (GO: 0043603), aromatic amino acid family metabolic processes (GO: 0009072), and lysine biosynthetic processes (GO: 0009085). The major categories representing cellular components were extracellular region parts (GO: 0044421), cytoskeletal parts (GO: 0044430), and cytoskeleton (GO: 0005856). Serine hydrolase activity (GO: 0017171), malic enzyme activity (GO: 0004470), endopeptidase activity (GO: 0004175), and hydrolase activity (GO: 0016641) were the most prominently represented GO terms in the molecular functions category (Table S6).

**Figure 6:**
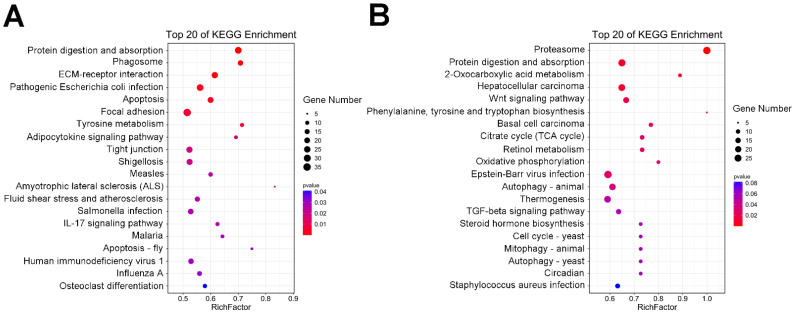
KEGG analysis of DEGs. (**A**) Circular graph of initial vs. black. (**B**) Circular graph of initial vs. blue.

The KEGG pathways that were significantly enriched in each of the DEG sets are shown in Table S7. As shown in Figure 6, the significantly enriched pathways (*p* < 0.05) in the initial vs. black, initial vs. blue, and white vs. black groups were involved in melanogenesis, including tyrosine metabolism (ko00350, Figure 6A); the Wnt signaling pathway (ko04310); phenylalanine, tyrosine, and tryptophan biosynthesis (ko00400, Figure 6B); and the cAMP signaling pathway (ko04024, Figure S2B). 

**Figure 7:**
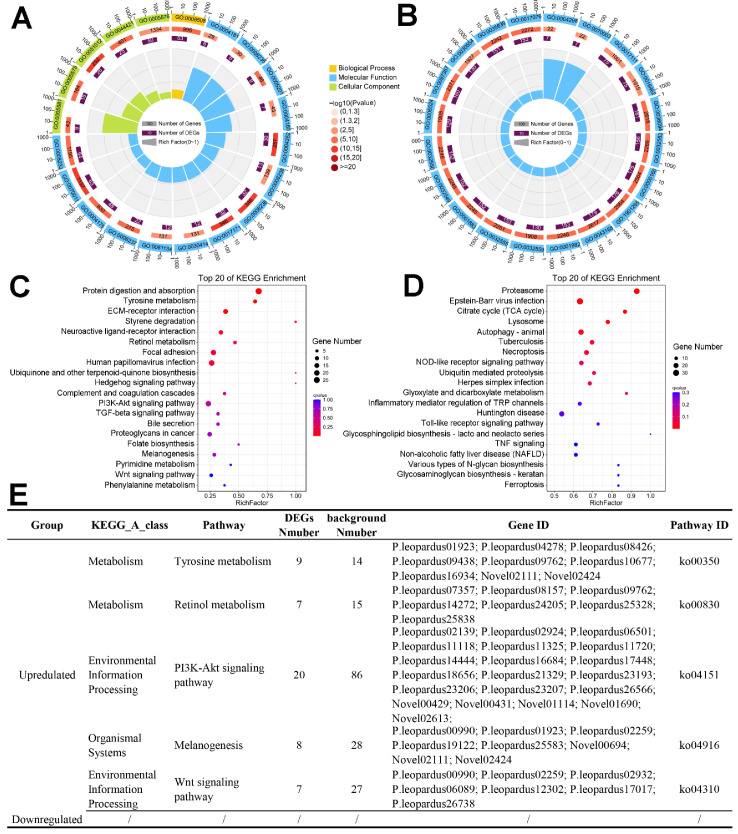
GO and KEGG enrichment results of DEGs. (**A**) GO terms of upregulated genes. (**B**) 20 GO terms of downregulated genes. (**C**) KEGG pathways of upregulated genes. (**D**) KEGG pathways of downregulated genes. (**E**) Melanogenesis-related pathways.

GO and KEGG analyses were performed to determine the enriched pathways of skin darkening genes in four groups of *P. leopardus* (Figure 7). A total of 471 GO terms were enriched in the DEG sets with a cutoff of *p* < 0.05 (Table S8). Figure 7A,B show the top 20 GO terms for the up- and downregulated genes, respectively. The major categories for biological processes in the upregulated group included multicellular organism development (GO: 0007275), developmental processes (GO: 0032502), and aromatic amino acid family catabolic processes (GO: 0009074). The cellular components terms were extracellular matrix (GO: 0031012) and proteinaceous extracellular matrix (GO: 0005578). The biological processes mainly involved serine-type peptidase activity (GO: 0008236), peptidase activity (GO: 0008233), endopeptidase activity (GO: 0004175), and L-tyrosine aminotransferase activity (GO: 0070547) (Figure 7A). The major biological processes categories in the downregulated group included transport (GO: 0006810) and cellular protein catabolic processes (GO: 0044257). The cellular components categories were proteasome complex (GO: 0031012) and cell junctions (GO: 0030054). The biological processes categories included small molecule binding (GO: 0036094), ATP binding (GO: 0005524), and hydrolase activity (Figure 7B).

The significantly enriched pathways in the KEGG analysis of upregulated genes were involved in melanogenesis (*p* < 0.05), including tyrosine metabolism (ko00350), the PI3K-Akt signaling pathway (ko04151), and retinol metabolism (ko00830) (Figure 7C). In addition, melanogenesis (ko04916), the Wnt signaling pathway (ko04310), phenylalanine metabolism (ko00360), and phenylalanine, tyrosine, and tryptophan biosynthesis (ko00400) were also enriched (*p* > 0.05) (Table S9). Figure 7E shows the upregulated genes in the key melanogenesis regulatory pathways. Many kinds of KEGG pathways were enriched with downregulated genes, such as the TCA cycle (ko00020), the NOD-like receptor signaling pathway (ko04621), the Toll-like receptor signaling pathway (ko04620), and alanine, aspartate, and glutamate metabolism (ko00250) (Figure 7D, Table S9).

### 2.6. qRT-PCR Verification

To further evaluate the reliability of RNA sequencing, 10 differentially expressed mRNAs (seven upregulated and three downregulated) were randomly selected to validate the relative expression levels in the dorsal skin by qRT-PCR. The mRNA primers are shown in Table S1. As shown in Figure S3, the qRT-PCR expression patterns of 10 genes were consistent with the RNA-seq results, confirming the reliability and accuracy of the RNA-seq-based transcriptome expression analysis.

## 3. Discussion

In teleost, color mutations are common, particularly in tropical ornamental fish. In order to reveal the molecular mechanisms and genetic basis of fish skin pigmentation, extensive studies have been conducted on model and commercially important fishes [6,19,20,23,24,25,26]. However, few studies have been conducted on the molecular mechanism of the effect of background on skin color. *P. leopardus* typically has red skin; however, the skin of most fish turns black under artificial culture conditions, affecting their commercial value [27]. In the present study, we examined the different skin colors of *P. leopardus* in response to different background colors by transcriptome analysis to investigate the molecular mechanism underlying the pigmentation abnormalities.

In the present study, the white group maintained a relatively red skin color compared to the initial group. Nevertheless, the skin color of the fish in the blue and black groups blackened, and the skin blackening is caused by the increase in the cell area of the melanophores. This is consistent with the findings for *C. auratus* [16,28], red tilapia [19], and other fish [8,9]. Previous studies have shown that bright light causes melanosomes to aggregate, leading to pale skin color, whereas dim light disperses the melanosomes [28]. In the present experiment, the white background tanks presented a bright underwater environment, the blue background tanks were intermediate, and the black background tanks were the darkest. Thus, background color affected the skin color of *P. leopardus* by adjusting the brightness of the water environment.

The variations in skin color were only an appearance, and the specific regulatory mechanism needs further exploration. We performed comparative transcriptome analysis among the initial, white, blue, and black groups of fish using Illumina sequencing technology, and 3179 DEGs were identified. Many DEGs were related to melanophores and melanin, such as *tyr*, *slc7a11*, *mc1r*, *ednrb*, *dct*, *tat*, and *wnt1* [20]. The *tyr* gene plays a role in melanin catalysis and synthesis, one of the downstream regulatory genes of melanocytes, associated with fish skin coloration and pigmentation [29]. In medaka and rainbow trout, the melanocytes become abnormal and skin color becomes orange and albino when the *tyr* sequence is mutated [30,31]. The *mc1r* gene is primarily expressed in the skin, and could influence the synthesis of melanin and dispersion of the melanosomes in melanophores [32]. The *slc7a11* gene directly acts on the dopaquinone to Cys-dopa pathway to maintain normal brown melanin synthesis in animals [33,34]. In our study, the expression of these genes increased as the skin darkened, indicating that these genes are also involved in the skin color darkening process in *P. leopardus*.

DEGs from six contrasting groups were generated and annotated by GO analysis. Then, GO analysis was conducted for the expression trends of the genes in the four groups after skin color blackening in *P. leopardus*. Most genes were involved in the biological processes category in all six sets, followed by molecular functions and cellular components (Table S5), such as amide biosynthetic processes (GO: 0043604), peptide metabolic processes (GO: 0006518), peptidase activity (GO: 0008233), and L-tyrosine aminotransferase activity (GO: 0070547). These results indicate that the variations in *P. leopard* skin color involve different biological functions.

The α-MSH/cAMP-dependent, MAPK, PI3K/Akt, and Wnt/β-catenin signaling pathways are key melanogenesis regulatory pathways, which activate or depress melanin synthesis [6,20]. In the present study, the significantly enriched pathways involved in melanogenesis included tyrosine metabolism (ko00350); the Wnt signaling pathway (ko04310); phenylalanine, tyrosine, and tryptophan biosynthesis (ko00400); and the cAMP signaling pathway (ko04024). These pathways were enriched in the initial vs. black, initial vs. blue, and white vs. black groups. Tyrosine metabolism (ko00350), the PI3K-Akt signaling pathway (ko04151), and the retinol metabolic (ko00830) pathways were also significantly enriched with upregulated genes (Table S9). In these pathways, the expression levels of some key genes related to melanin synthesis increased, such as *α-MSH*, *wnt*, *dct*, and *tyrp*. In teleosts, chronic treatment with α-MSH darkens body color [13,32]. The α-MSH peptide binds to *mc1r*, increasing intracellular cAMP levels, stimulating adenylyl cyclase and activating protein kinase A, which activates tyrosinase to produce melanin [32,35]. Injecting red tilapia with α-MSH in the caudal vein results in significantly higher tyrosinase activity and melanin content in the dorsal and ventral skin [36]. The *wnt* gene affects melanin synthesis by affecting the *mitf* expression level through *β-catenin* [19]. *tyrp* is mainly involved in the catalysis of melanin by maintaining the stability of *tyr* on the melanin membrane and inhibiting apoptosis of immature melanocytes [37].

**Figure 8:**
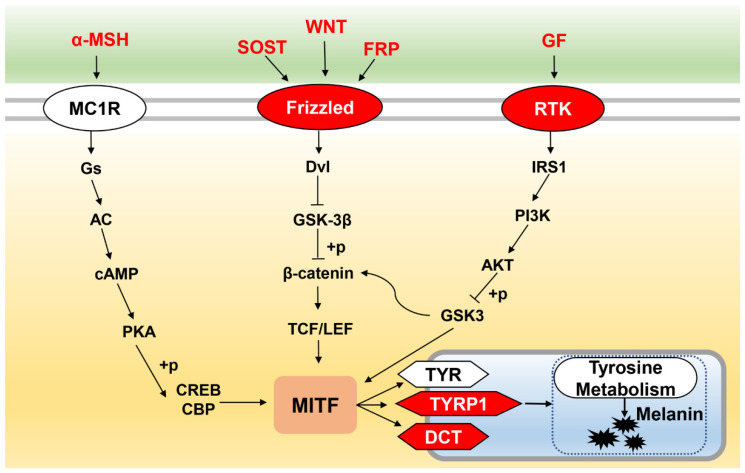
The hypothesis regarding the regulation mechanism of the effect of background color on skin color variation in *P. leopard*. Red words and white words on the red background means upregulated genes.

We hypothesized the regulatory mechanism of the effect of background color on the skin color variations in *P. leopardus*. The concrete genes and pathways are shown in Figure 8. As the background color deepens, the expression levels of some key genes are upregulated, such as *α-msh*, *wnt*, and *gf*. Then, the α-MSH/cAMP-dependent, Wnt/β-catenin, and PI3K/Akt signaling pathways are activated, resulting in the accumulation of nuclear *mitf*. *mitf* promotes the production of melanin by binding to the conserved M-box motif in the tyr/tyrp1/dct promoter region [6].

## 4. Materials and Methods

### 4.1. Experiment, Fish Sampling, and Tissue Preparation

The experimental fish were obtained from Dongfang Star Technology Co., Ltd. (Ledong, China). Juvenile fish were about 10 g at 4 months post-hatch. Nine experimental aquaria with circulating water (length × width × depth = 60 × 40 × 40 cm) were prepared, and they were pasted with white, blue, and black polypropylene plastic board. Fishing nets were placed over the aquaria to prevent the fish from escaping. The juvenile *P. leopardus* were randomly divided into three groups of blue, black, and white, with 30 fish per aquarium and three replicates per group. The fish were reared in the indoor aquaria for 56 days under a 12 h light/dark cycle at 28 ± 1 °C and fed a commercially prepared diet (Guangdong Yuequn Biotechnology Co., Ltd., Jieyang, Guangdong Province, China) to satiation three times daily at approximately 08:00, 12:00, and 17:00.

Photographs of the fish were taken at the beginning and end of the experiment. Skin tissue was collected, washed with normal saline, prepared into temporary pack pieces, and photographed on a Stemi 508 stereo microscope. Dorsal skin tissues were collected from 48 fish (12 fish from the initial, white, blue, and black groups, respectively). Three fish samples were pooled into one microcentrifuge tube (nine replicates per group), snap-frozen in liquid nitrogen, and stored at −80°C until processed.

### 4.2. Total RNA Extraction and Qualification

Total RNA was extracted from the dorsal and ventral skin using TRIZOL reagent (Invitrogen, Carlsbad, CA, USA) according to the manufacturer’s protocol. Genomic DNA was removed from the RNA samples using DNase I (New England Biolabs, Ipswich, MA, USA). Total RNA was qualified and quantified by 1% agarose gel electrophoresis, the NanoPhotometer^®^ spectrophotometer, and the RNA Nano 6000 Assay Kit. The isolated RNA samples were dissolved in RNase-free water and stored at −80 °C.

### 4.3. cDNA Library Construction and Sequencing

Twelve cDNA libraries (three pooled samples per group) were constructed with 12 RNA samples (the OD 260/280 ratios were 2.0–2.2, the OD 260/230 ratios were >1.5, and the RNA Integrity Number was >8.0). After preparing the libraries and pooling different samples, the samples were subjected to Illumina sequencing. The libraries were sequenced on the Illumina Novaseq 6000 Platform and generated 150 nt paired-end reads. The RNA-seq library was prepared and sequenced by Novegene Technology Co., Ltd. (Beijing, China).

### 4.4. Quality Control, Mapping, and Assembly

The FastQC was used for quality control and the read statistics [38]. Clean and high-quality reads were collected for all downstream analyses, and Q20, Q30, and GC contents were calculated. HISAT2 software and the *P. leopardus* reference genome were used for clean read mapping [39]. The transcripts were assembled by the StringTie program [40]. 

### 4.5. Differently Expressed Genes (DEGs) Analysis

The read numbers mapped to each gene were counted using HTSeq v0.6.1, and the reads per kilobase of transcripts per million mapped reads (RPKM) were obtained. The differently expressed genes were analyzed by R package. Genes with |log2 (fold change)| > 1 and adjusted *p*-values < 0.05 were defined as differentially expressed. The Benjamini and Hochberg approach was used to adjust *p*-values to control the false discovery rate.

### 4.6. Functional Annotation and Pathway Enrichment

The GOseq R package was used for Gene Ontology (GO) enrichment analysis of the DEGs. Corrected *p*-values < 0.05 were considered significantly enriched. The statistical enrichment of the DEGs in the KEGG pathways was tested by KOBAS software. The significantly enriched KEGG pathways related to our study, including those related to pigment, tyrosine metabolism, and melanin biosynthesis, were selected for further analysis. The focal pathways were integrated by the KEGG database [41].

### 4.7. Quantitative Real-Time Polymerase Chain Reaction (qRT-PCR) Validation

To determine the accuracy of the sequencing data, the DEGs involved in the color separation between fish living in light and dark backgrounds were selected for qRT-PCR. The Primer Premier 5 software was used to design primers (Table S1). The amplification reactions containing 1 μL of cDNA, 1 μL of each primer (10 μM), 12.5 μL of 2 × SYBR Green MasterMix reagent, and 9.5 μL of PCR-grade water, in a total volume of 25 μL, were performed on the ABI PRISM 7500 Real-time PCR System. The thermal cycling profile was 5 min at 95 °C for initial denaturation, followed by 40 cycles of 15 s at 95 °C for denaturation and 45 s at 60 °C for annealing/extension. The *β-actin* gene served as an internal control, and the 2^−ΔΔCT^ method was used to analyze the relative expression levels of DEGs [42].

### 4.8. Statistical Analysis

The SPSS version 22.0 software (SPSS Inc., Chicago, IL, USA) was used for data analysis, and all data are presented as mean ± standard error. All data were analyzed by one-way analysis of variance after the homogeneity of variance test. A *p*-value < 0.05 was considered significant. Duncan’s multiple range test was used to detect differences between the experimental groups. Differences between two groups were detected using Student’s *t*-test.

## 5. Conclusions

In this study, we performed a comparative transcriptome analysis among four different color types of *P. leopardus* exposed to different background colors. The RNA-seq analysis yielded 26,675 known mRNAs, 3278 novel mRNAs, and 3179 DEGs. The DEGs related to melanin synthesis were screened out. Some melanin-related candidate genes were *tyr*, *slc7a11*, *mc1r*, *ednrb*, *dct*, *tat*, and *wnt1*. These DEGs were mainly involved in melanogenesis, including tyrosine metabolism, the Wnt signaling pathway, and the cAMP signaling pathway. We hypothesized about the regulatory mechanism of the effect of background color on the skin color variations in *P. leopardus*.

## Data Availability

All data generated and analyzed during this study are included in the published article. The original data has been uploaded to the NCBI_GEO database, NCBI_SRA accession no. PRJNA849384.

## Supplementary Materials

The following supporting information can be downloaded at: https://www.mdpi.com/article/10.3390/ijms231911186/s1.
