# Supplementary material for: Transcriptome Analysis Reveals the Complex Regulatory Pathway of Background Color in Juvenile Plectropomus leopardus Skin Color Variation
Source: Int J Mol Sci. 2022 Sep 23;23(19):11186. doi: 10.3390/ijms231911186 (PMC9569894; doi:10.3390/ijms231911186)
Supplement: Supplementary file 1 [file ijms-23-11186-s001.zip › Figure and Table Caption.pdf]

Figure S1: GO analysis of DEGs. (A) Circular graph of initial vs. white. (B) Circular graph of initial vs. black. (C) Circular graph of initial vs. blue. (D) Circular graph of white vs. black. (E) Circular graph of blue vs. white. (F) Circular graph of blue vs. black;
Figure S2: KEGG analysis of DEGs. (A) Circular graph of initial vs. white. (B) Circular graph of white vs. black. (C) Circular graph of blue vs. white. (D) Circular graph of blue vs. black;
Figure S3: Validation of RNA-seq expression patterns using qRT-PCR

Table S1: Primers used for qRT-PCR; Table S2: Alignment of the statistical results of the reads; Table S3: Characterization of these transcripts in *P. leopardus* skin; Table S4: The different gene expression profiles of six contrast groups; Table S5: Expression levels of differentially expressed mRNAs; Table S6: GO enrichment analyses of differentially expressed mRNAs; Table S7: KEGG enrichment analyses of differentially expressed mRNAs; Table S8: GO enrichment analyses of trends of expressed mRNAs; Table S9: KEGG enrichment analyses of trends of expressed mRNAs.
