# Supplementary figures and images for: Transcriptome Analysis Reveals the Complex Regulatory Pathway of Background Color in Juvenile Plectropomus leopardus Skin Color Variation
Source: Int J Mol Sci. 2022 Sep 23;23(19):11186. doi: 10.3390/ijms231911186 (PMC9569894; doi:10.3390/ijms231911186)

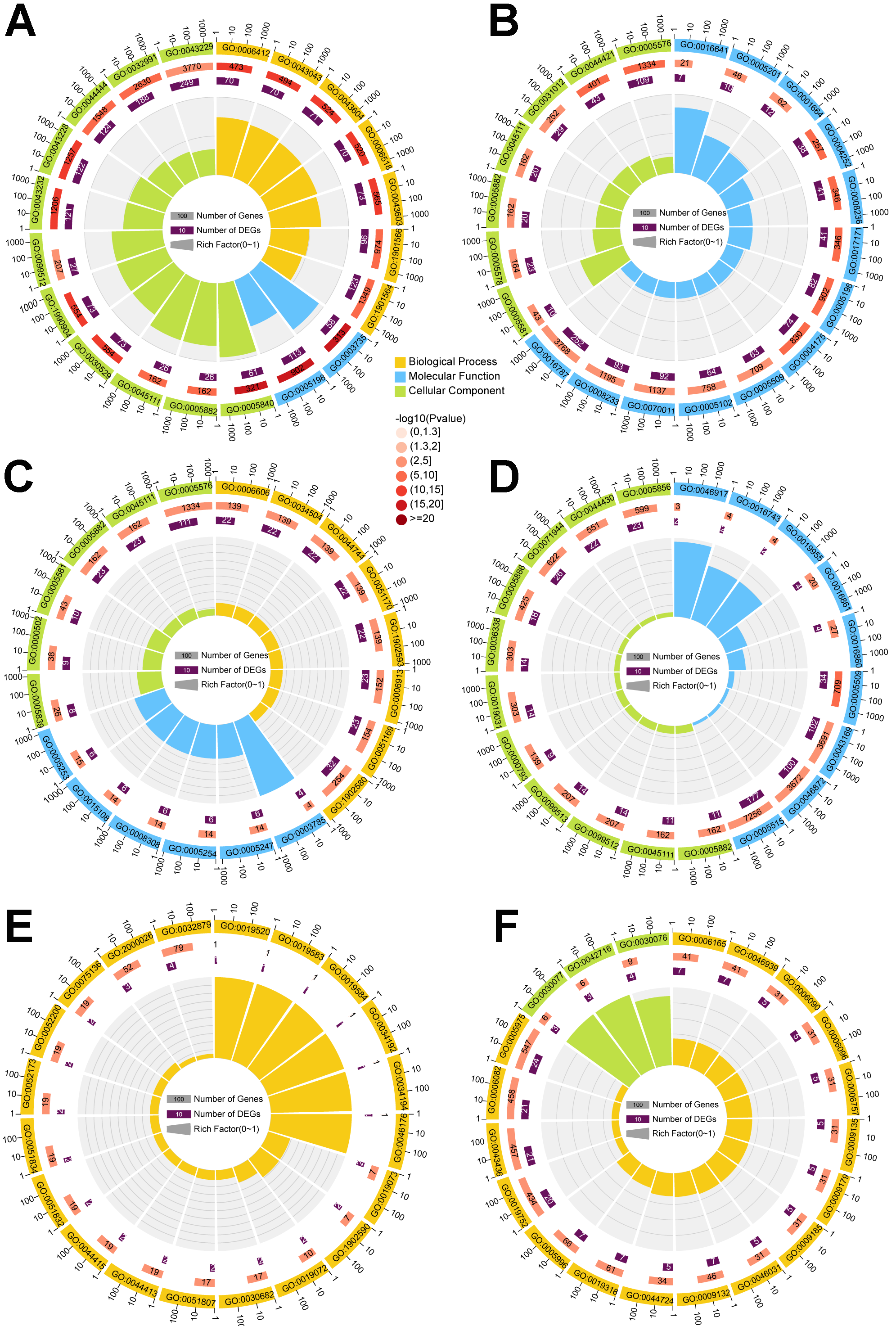

Supplement: Supplementary file 1 [file ijms-23-11186-s001.zip › figure S1.tif]

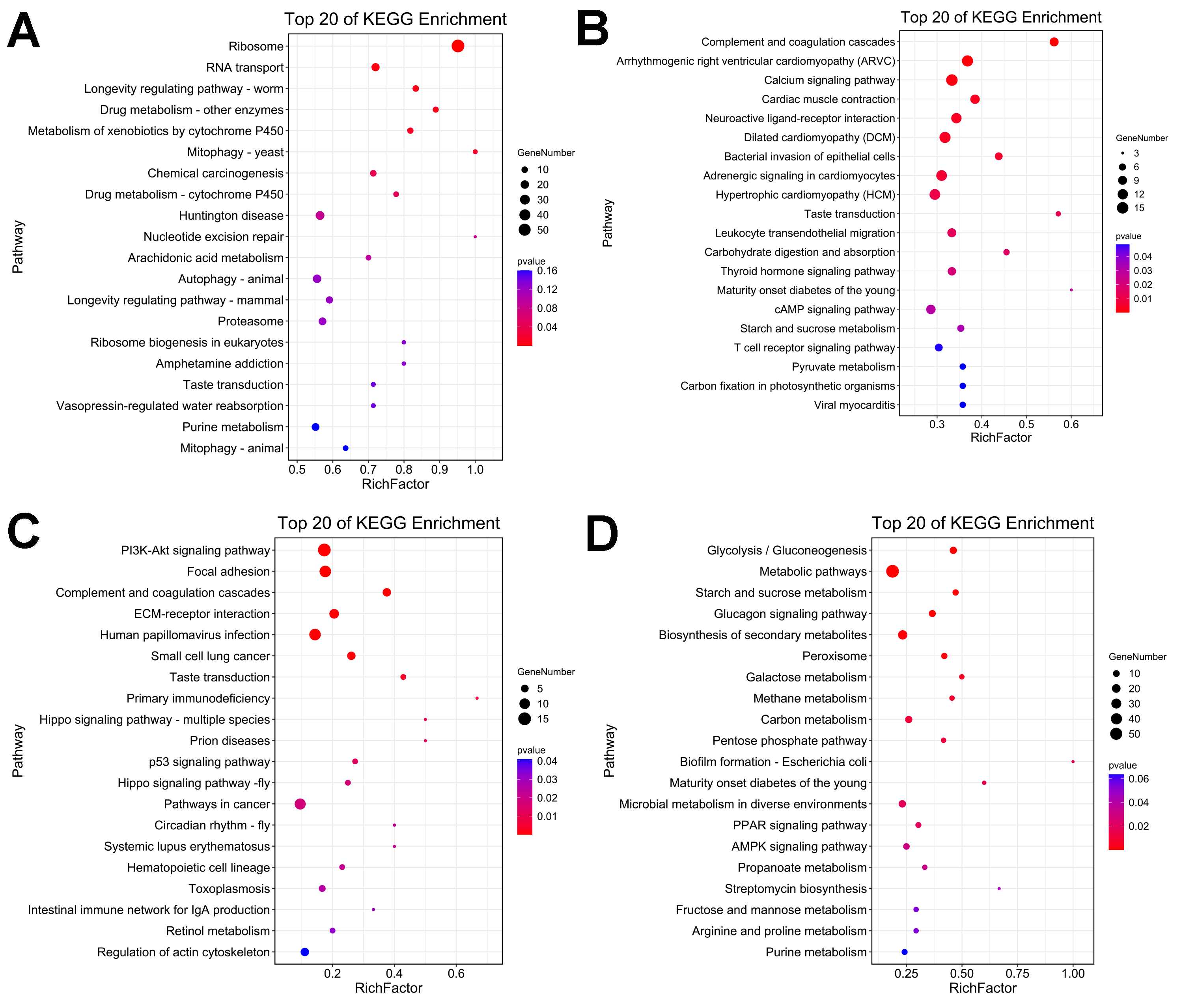

Supplement: Supplementary file 1 [file ijms-23-11186-s001.zip › figure S2.tif]

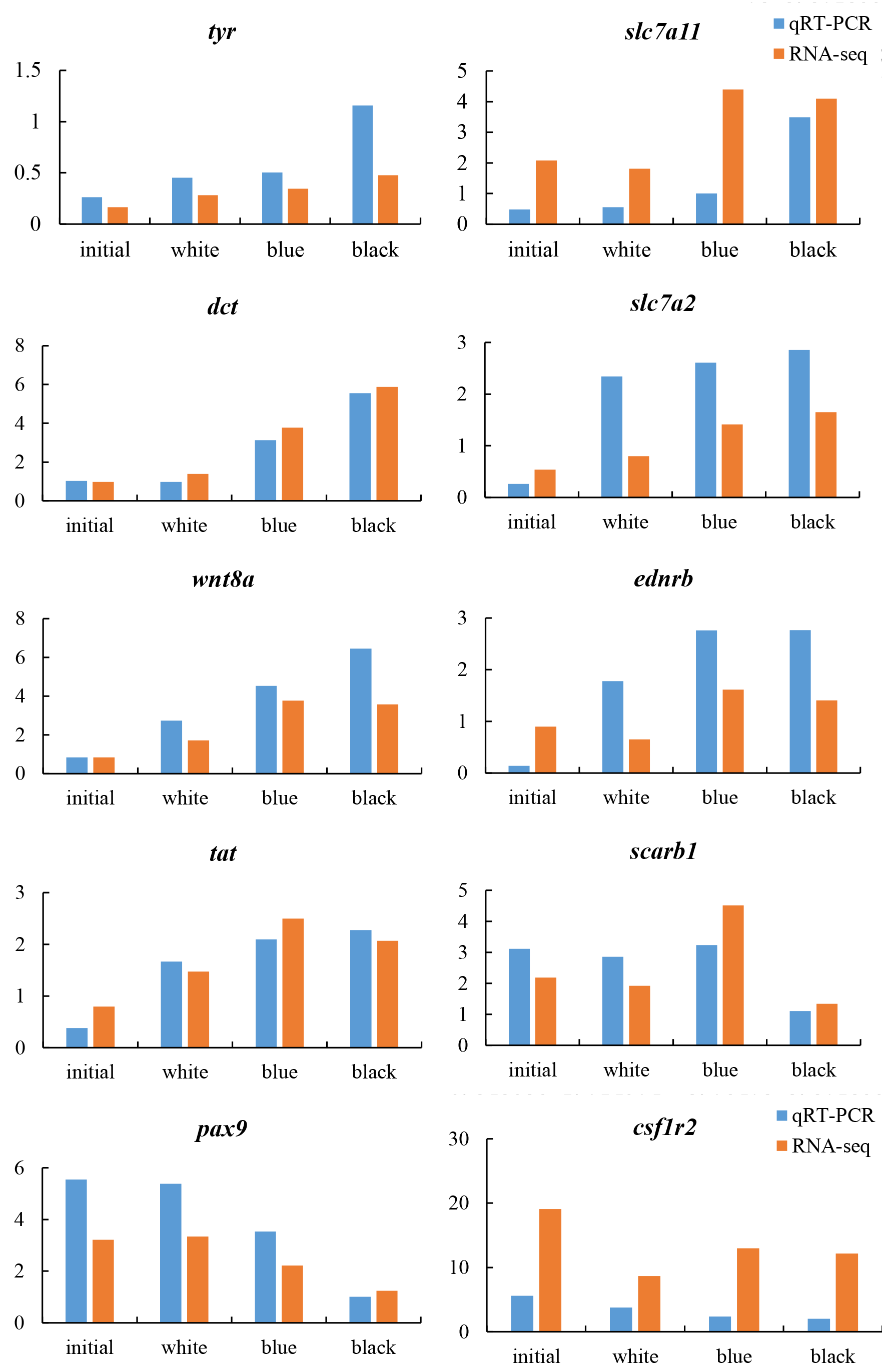

Supplement: Supplementary file 1 [file ijms-23-11186-s001.zip › Figure S3.tif]
